# Supplementary material for: A community developed conceptual model for reducing long-term health problems in children with intellectual disability in India
Source: PLOS Glob Public Health. 2023 Apr 14;3(4):e0000833. doi: 10.1371/journal.pgph.0000833 (PMC10104324; doi:10.1371/journal.pgph.0000833)
Supplement: S1 File — (DOCX) [file pgph.0000833.s001.docx]

**Guidance for using and contextually adapting the conceptual model**

**F**amily-centric **I**nclusive **I**ntervention for **C**hildren with **I**ntellectual disability involving the **C**ommunity and **C**ross **S**ectoral **S**ystem (**FICCSS**)

The conceptual model consists of three inter-related parts:

1. Community-based, cross-sectoral, family-centred inclusive intervention.
2. Factors that will facilitate uptake and participation by the target beneficiaries: children with intellectual disability (ID) and their parents/caregivers.
3. Individual, family, societal and policy level factors that would contribute to the impact of the intervention.

**1. Community-based, cross-sectoral, family-centred inclusive intervention**

The intervention has five evidence-based core components, which can be adapted according to context and needs:

1. A physical activity programme for the children.
2. A guide to a healthy diet to provide good nutrition to the children using locally available and affordable food.
3. Encouraging and supporting parents to incorporate adaptive behavioural and cognitive stimulation, including in everyday activities alongside other children who do not require specialist resources.
4. Education and training to improve parent’s knowledge of accessing healthcare services.
5. Group counselling for parents and children to increase their capability to address behavioural issues, individual barriers to exercise and how to follow a healthy diet, improve emotional and mental wellbeing, and to encourage social inclusion.

***Target age group for children with ID for the intervention***

Our recommendation is to start the health promotion activities from an early age, 4-10 years, to build on the “Early Intervention Services” for all children 0-3 years of age available in many countries. This will ensure early identification of barriers, support continuity and facilitate behaviour change from an early age to address some of the modifiable risk factors by promoting a healthy lifestyle, prevent under and over nutrition, improve healthcare access for children, and reduce stress and anxiety among the children and their parents.

**2. Factors that will facilitate uptake and participation**

The intervention components should be contextually relevant to the local culture and context, and where possible embedded within the day-to-day activities of the children. They should be simple to deliver and flexible according to local circumstances. Guidance for adapting the components to different contexts is summarised in Table 1 below. Adaptations should pro-actively involve children with ID and their parents, and professionals working and supporting children with ID.

**Table 1: Guidance for contextual adaptation of the framework**

| Intervention component | Content of the evidence- based intervention | Examples of contextual adaptation of the contents *(based on stakeholder recommendations)* |
| --- | --- | --- |
| 1. PHYSICAL ACTIVITY | Exercise and sports for the children*.* | Adaptations could be made to align with the day to day activities of the children (e.g. walking to school, village shop; helping at home, inclusive community sports). Inclusion could be promoted by embedding the intervention with activities for other children. |
| 2. DIET & NUTRITION | Healthy diet using locally available food. Parents and children advised on how they could swap unhealthy foods with healthy food choices, for example swapping sweets with fruits. | Locally available food items that are generally consumed by children could be categorised as good or bad for their health, and how food availability and socio-economic status influence food choices should be examined. *Bad* food choices could be mapped with a range of available and affordable food items containing good nutrition to recommend swapping. |
| 3. CONTIUED SUPPORT – ADAPTIVE, BEHAVIOURAL AND COGNITIVE STIMULATION | Continued support to maintain behavioural and cognitive stimulation through regular concentration and attention/ behavioural enhancement tasks supported by parents. | Cognitive stimulation alongside support and development of adaptive behaviour to support children’s independence in practical, social and conceptual skill (example: functional skills such as personal care, social skills, home living).  Support for parents in how to break down day to day tasks to enable children to do them as well as encouragement and praise, alongside siblings and peers. |
| 4. HEALTHCARE | Education and training to improve parent’s knowledge of accessing healthcare services.  Education and training of healthcare providers on inclusive provision. | Understand parents’ perception of accessibility and inclusiveness of services and the barriers to access to guide the contents of this intervention component. For example, it is possible that structural barriers and behaviour or attitudes of health professionals influence care seeking rather than the attitude of parents per se. |
| 5. GROUP COUNSELLING AND SOCIAL INCLUSION | Group counselling and peer-support to discuss and improve emotional and mental wellbeing of parents. Encourage parents to promote social inclusion along with community members and professionals. | Peer-support groups should be organized with some professional support and counselling.  Behavioural outcome measures and targets should be set individually (based on the baseline assessment) and should be reviewed throughout the course of the intervention. |

**3. Factors that would influence the impact of the intervention**

The model clearly shows that the cross-sectoral family-centred programme is not a standalone intervention, but will need to be supported by an inclusive enabling environment to address the stigma and social exclusion that exacerbate the course and experience of health problems by children with ID. For the intervention to be successful, it is essential to identify and mitigate social and health system barriers, and actively promote community mobilisation to integrate the intervention within existing health and social care services. This will require attention to the structural, social and health system barriers, especially those related to stigma that limit inclusion.

Some examples of how these might be addressed include:

- Organise public awareness campaigns through audio-visual and social media about disability and inclusion to reduce stigma and improve inclusion;
- Change the medical education curriculum to facilitate inclusion of ‘disability-inclusive compassionate care’ and ‘disability rights’, which can contribute to removing health system barriers;
- Implement regional and national policy actions to facilitate inclusion of children with ID into the mainstream health promotion activities in order to align with the sustainable and inclusive approach recommended by the United Nations Convention on the Rights of Persons with Disabilities (UNCRPD) and the Sustainable Development Goals (SDGs).

***Who can implement the intervention?***

Health promotion for children with ID can be implemented through the inclusive education programme, but we recommend that care providers from a range of public and private sectors be involved, as such educational programmes may not be available or accessible to all children. Care providers employed by government and non-government institutions, such as the health system, nutrition and social welfare sectors and schools for children with disabilities would be better placed to deliver the intervention due to their understanding of the children’s complex needs. Where available, local disability rehabilitation centres could coordinate the delivery of the intervention and follow-up. Trained workers in such centres are already working towards improving societal awareness and attitudes, community inclusion of people with disability, and school enrolment, and helping families access services, benefits, and support for their children.
