# Supplementary material for: A community developed conceptual model for reducing long-term health problems in children with intellectual disability in India
Source: PLOS Glob Public Health. 2023 Apr 14;3(4):e0000833. doi: 10.1371/journal.pgph.0000833 (PMC10104324; doi:10.1371/journal.pgph.0000833)
Supplement: Attachment — Submitted filename: Response letter.docx [file pgph.0000833.s003.docx]

**Response to reviewer’s comments**

**Addressing Journal requirements**

1. Please review your reference list to ensure that it is complete and correct.
   **We have reviewed the reference list and can confirm that we do not have any retracted papers in the reference list. Only reference 6 was updated using the up-to-date reference for the paper. The list is complete and correct to the best of our knowledge.**
2. Please include a complete copy of PLOS’ questionnaire on inclusivity in global research in your revised manuscript.
   **The filled questionnaire is included as supplementary material, as advised.**
3. Please insert an Ethics Statement at the beginning of your Methods section, under a subheading 'Ethics Statement'.
   **As recommended, we have moved the ‘ethics statement’ from the end to the beginning of the methods section.**
4. Statement on data availability.
   **An exemption was requested and approved before the paper was accepted for review by the Journal.**

**Response to reviewer’s comments**

Reviewer #1: This project highlights the plight of a neglected section of society in one of the world's most populous countries. The authors have consulted widely and involved the consultees in reviewing the Theory of Change model they have jointly developed. The authors describe their conceptual framework to target the five evidence based core components for intervention.

**Response:** We thank the reviewer for the positive and encouraging comments.

The Theory of Change model is not clearly described. Figure 2 is inevitably complex and crowded and the paragraph between lines 297 - 312 needs to be revised to highlight the components of the ToC.

**Response:** We agree with the reviewer that there is a need to describe the complex Theory of Change model. We have added further details in the revised manuscript on pages 13-14, lines 311-323 in the marked copy.

The ToC mentions the "accountability ceiling" and the "Transfer of ownership" but there is no elaboration of the interface between local and national stakeholders. Without this being clarified it is likely that the worthwhile issues put forward will not translate into policy and sustained action which this section of the population is crying out for.

**Response:** We thank the reviewer for raising this important point. As advised, we have added more information to elaborate how we plan to achieve the transfer of ownership and accountability from the local to the national bodies for a sustainable impact. Please see revised manuscript, pages 14-15, lines 333-343 in the marked copy.
