# Supplementary material for: A community developed conceptual model for reducing long-term health problems in children with intellectual disability in India
Source: PLOS Glob Public Health. 2023 Apr 14;3(4):e0000833. doi: 10.1371/journal.pgph.0000833 (PMC10104324; doi:10.1371/journal.pgph.0000833)
Supplement: Attachment — Submitted filename: Response to reviewers comments_R2.docx [file pgph.0000833.s004.docx]

**Response to reviewers’ comments**

Journal Requirements:

1. Please review your reference list to ensure that it is complete and correct. If you have cited papers that have been retracted, please include the rationale for doing so in the manuscript text, or remove these references and replace them with relevant current references. Any changes to the reference list should be mentioned in the rebuttal letter that accompanies your revised manuscript. If you need to cite a retracted article, indicate the article’s retracted status in the References list and also include a citation and full reference for the retraction notice.

Response: We have reviewed the reference list and can confirm that we do not have any retracted papers in the reference list. The list is complete and correct to the best of our knowledge.

2. We have noticed that you have uploaded Supporting Information files, but you have not included a list of legends. Please add a full list of legends for your Supporting Information files after the references list.

Response: We have now added a list of legends after the reference list in the revised manuscript (lines 616-618 of the revised manuscript in track changes).

Reviewer's Responses to Questions

**Comments to the Author**

1. If the authors have adequately addressed your comments raised in a previous round of review and you feel that this manuscript is now acceptable for publication, you may indicate that here to bypass the “Comments to the Author” section, enter your conflict of interest statement in the “Confidential to Editor” section, and submit your "Accept" recommendation.

Reviewer #1: All comments have been addressed

Reviewer #2: All comments have been addressed

Response: We thank the reviewers for their comments and are pleased to know that we addressed all comments from the previous round.

6. Review Comments to the Author

Please use the space provided to explain your answers to the questions above. You may also include additional comments for the author, including concerns about dual publication, research ethics, or publication ethics. (Please upload your review as an attachment if it exceeds 20,000 characters)

Reviewer #1: thank you responding to my earlier comments

Response: We thank the reviewer for helping us improve the paper.

Reviewer #2: The authors of this manuscript have done great job for their effort and energy put into this work. The subject matter of the manuscript is critical for the development and well-being of children with intellectual disability in India and other parts of the work where the conceptual framework and model can be replicated to develop and implement interventions for children with intellectual disability.

Response: We thank the reviewer for the positive comments and for the new suggestions made in this round to further strengthen the manuscript.

There is a clarification that I think needs to be made to strengthen the manuscript even more. That is the methodology section of this manuscript. Indeed, you have comprehensively described the consultative meetings and interviews with the various stakeholders. However, readers will still need to understand how these numerous stakeholders were sampled and what were the nature of the interviews and discussions conducted. Please, provide the inclusion and exclusion criteria for selecting various stakeholders from the institutions and communities.

Response: As advised by the reviewer, we have clarified the inclusion and exclusion criteria in the revised manuscript on page 5, lines 112-119.

The second issue I will like o see you improve is the diagram of conceptual framework. No doubt you did a wonderful job but it framework appears a little complex and needs to be simplified for the healthcare and educational instructors who will developing programs based on it. I will also recommend that you come out with some form of guidelines on how to use the model.

Response: As advised by the reviewer we have simplified the conceptual framework (Figure-1) included in the revised manuscript. We have developed a short guideline to facilitate the use of the model, and will make this available through our institutional website and partner organisations. Please see additions on page 19, lines 449-451 of the revised manuscript in track changes, and the supplementary file.

After reading the manuscripts many times, it sounds to me that the manuscript is a report of consultative discussions rather than a typical research article. This is more so you indicated this type of work is exempt from ethical review and approval. Please, clarify.

Response: We agree with the reviewer that this is not a typical research article, although we submitted it under the “original research” category of the Journal. We have further clarified the ethics section at the beginning of the ‘Methods’ (page 4, lines 78-94 in the tracked version).

Despite the above, this work is undoubtedly comprehensive, fully participatory and well presented.

Response: We thank the reviewer again for their time and for the helpful comments and suggestions.
